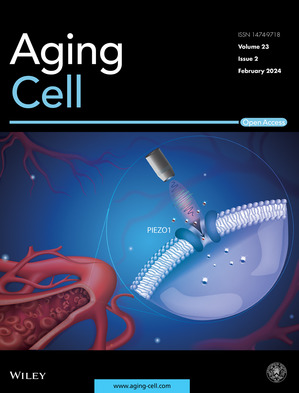# Featured Cover

**DOI:** 10.1111/acel.14116

**Published:** 2024-02-12

**Authors:** Ngoc Luu, Apratim Bajpai, Rui Li, Seojin Park, Mahad Noor, Xiao Ma, Weiqiang Chen

## Abstract

Cover legend: The cover image is based on the Research Article *Aging‐associated decline in vascular smooth muscle cell mechanosensation is mediated by Piezo1 channel* by Ngoc Luu et al., https://doi.org/10.1111/acel.14036